# Anti-Hepatitis B Virus Activity of Esculetin from *Microsorium fortunei* In Vitro and In Vivo

**DOI:** 10.3390/molecules24193475

**Published:** 2019-09-25

**Authors:** Si-Xin Huang, Jun-Fei Mou, Qin Luo, Qing-Hu Mo, Xian-Li Zhou, Xiao Huang, Qing Xu, Xiang-Duan Tan, Xu Chen, Cheng-Qin Liang

**Affiliations:** 1 College of Pharmacy, Guilin Medical University, Guilin 541004, Guangxi, China; 2 Biotechnology Institute, Guilin Medical University, Guilin 541004, Guangxi, China; 3 Science Experiment Center, Guilin Medical University, Guilin 541004, Guangxi, China; 4 Guangxi Key Laboratory of Molecular Medicine in Liver Injury and Repair, Guilin Medical University, Guilin 541004, Guangxi, China

**Keywords:** Hepatitis B virus, Esculetin, *Microsorium fortunei*, HepG2.2.15, duck Hepatitis B virus

## Abstract

Coumarins are widely present in a variety of plants and have a variety of pharmacological activities. In this study, we isolated a coumarin compound from *Microsorium fortunei* (Moore) Ching; the compound was identified as esculetin by hydrogen and carbon spectroscopy. Its anti-hepatitis B virus (HBV) activity was investigated in vitro and in vivo. In the human hepatocellular liver carcinoma 2.2.15 cell line (HepG2.2.15) transfected with HBV, esculetin effecting inhibited the expression of the HBV antigens and HBV DNA in vitro. Esculetin inhibited the expression of Hepatitis B virus X (HBx) protein in a dose-dependent manner. In the ducklings infected with duck hepatitis B virus (DHBV), the levels of DHBV DNA, duck hepatitis B surface antigen (DHBsAg), duck hepatitis B e-antigen (DHBeAg), alanine aminotransferase (ALT) and aspartate aminotransferase (AST) decreased significantly after esculetin treatment. Summing up the above, the results suggest that esculetin efficiently inhibits HBV replication both in vitro and in vivo, which provides an opportunity for further development of esculetin as antiviral drug.

## 1. Introduction

The hepatitis B virus (HBV) is a common infectious disease in the world. According to reports, there are about 350 million hepatitis B virus carriers worldwide [1,2]. There are a series of figures showing that about one million people worldwide die from complications caused by HBV every year [3], which poses a great threat to human health. At present, the drugs commonly used for anti-HBV are interferon and nucleosides such as IFN-α, lamivudine (3TC), tenofovir, etc. However, these drugs are prone to drug resistance and have side effects [4]. Thus far, special drugs and methods of treating hepatitis B have not been discovered [5,6]. In the last few years, much clinical and experimental research has verified many Chinese medicines with anti-HBV effects [7]. Therefore, it is a hot topic in the search for new drugs to find high-efficiency, low-cost and novel anti-HBV drugs from traditional Chinese herbal medicine.

*Microsorium fortunei* (T.Moore) Ching is a genus of Microsorium, belonging to the family of Polypodiaceae, and its whole grass and rhizome can be used as medicine. In addition to being a Tujia medicine, the botanical drug is used to treat stomach cold pain, bronchial asthma, rheumatic pain, gonococcal disease and dysentery [8], and is also used by other ethnic minorities of Miao, Mulao and Lisu nationality. It is also used for pain of body surface or internal organs and snake bite, scorpion venom, burns, etc. [9]. By consulting the literature, it was found that esculetin could inhibit tumor cells [10,11,12], enhance the body′s defense ability and inhibit the expression of inflammatory cytokines [13]. It has also been reported that esculetin can improve liver injury [14,15]. A series of studies have used mice to construct a liver injury model and found that esculetin has the effect of improving liver damage [16,17]. From the above, there have been a few studies on the substance’s anti-HBV activity. The aim of this study was to isolate the esculetin from *Microsorium fortunei* and to use the duckling model for the first time to study the hepatic injury of esculetin, and to explore the anti-HBV mechanisms.

Viruses often encode proteins that regulate normal cellular processes to create an environment conducive to viral replication. HBx is a viral protein with multiple regulatory functions encoded by the X open reading frame of hepatitis B virus [18]. Studies have shown that HBx protein can regulate the expression of a variety of host genes [19]. HBx directly or indirectly targets viruses by activating the transcription of host cells and the virus′s own genes, regulating apoptosis, inhibiting the repair of damaged DNA in cells, and activating signal transduction pathways such as mitogen-activated protein kinase (MAPK) and c-Jun N-terminal kinase (JNK) in cells. It has a wide-ranging effect on its own replication and proliferation, as well as the expression and regulation of genes in host cells, apoptosis and carcinogenesis [20]. HBV replication levels in HepG2.2.15 cells are lower in S phase, but higher in G_0_/G_1_ phase [21]. When other proteins of HBV are deleted, HBx expression can induce normal hepatocytes from the G_0_ phase to the G_1_ phase and reduce the levels of p15 and p16 (two proteins inhibit the cell cycle from G_0_ to G_1_), increasing the levels of G_1_ phase proteins P21, P27, cyclin D1 and cyclin E. Gearhart et al. [22] found that HBx protein-induced quiescent hepatocytes entering G_1_ phase are essential for HBV replication, and regulation of hepatocyte proliferation pathway is a basic function of HBx-activated HBV replication.

Many studies have indicated that duck hepatitis B virus (DHBV) and HBV are similar in biological characteristics, morphological structure, nucleic acid composition and causing host liver disease. Ducks are easily infected with HBV; the benefits for clinical studies are that there are many of them, and they are easy to raise. Therefore, the duck hepatitis B animal model is a model of widely using to study hepatitis B [23]. To explore the anti-hepatitis B virus effect of esculetin, a congenitally infection model of duck hepatitis B was used to explore the effect of different doses of esculetin on DHBV.

## 2. Results

### 2.1. Structural Identification

**Figure 1:**
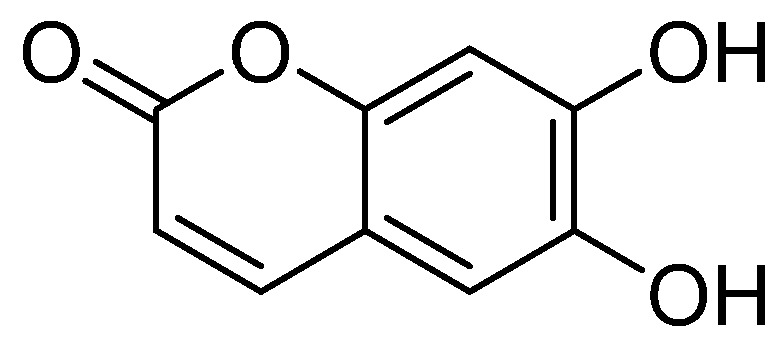
Chemical structure of esculetin.

The compound is a yellow amorphous powder. Electrospray ionization mass spectrometry (ESI-MS) *m/z*: 177[M − H]^−^. ^1^hydrogen-nuclear magnetic resonance (^1^H-NMR)(500 MHz, dimethylsulfoxide, DMSO-*d*_6_) δ: 7.85 (1H, d, *J* = 9.5 Hz, H-4), 6.99 (1H, s, H-5), 6.76 (1H, s, H-8), 6.14 (1H, d, *J* = 9.5 Hz, H-3); ^13^Carbon-NMR (^13^C-NMR) (125 MHz, DMSO-*d*_6_) δ:160.9 (C-2), 111.4 (C-3), 144.5 (C-4), 112.3 (C-5), 143.1 (C-6), 150.6 (C-7), 102.7 (C-8), 148.5 (C-9), 110.6 (C-10). Based on these data [24], the compound was identified as esculetin. Its molecular formula and molecular weight are C_9_H_6_O_4_ and 178, respectively. The chemical structure of esculetin is shown in Figure 1. Based on the High Performance Liquid Chromatography (HPLC) analysis (Figure S1), the purity of esculetin was greater than 98%. The ^1^H-NMR and ^13^C-NMR spectra of the compound are shown in Figure S2, and Figure S3, respectively.

### 2.2. Cytotoxic Effect of Esculetin for HL-7702 Cells

**Figure 2:**
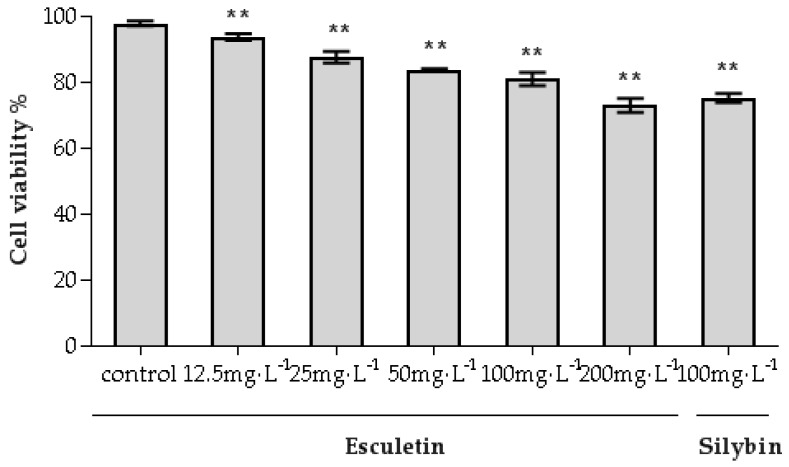
The inhibitory effect of esculetin for HL-7702 cells (* *p* < 0.05, ** *p* < 0.01).

The cytotoxicity of esculetin for HL-7702 cells was determined by the trypan blue assay [25,26,27]. As shown in Figure 2, after the sample was applied to the cells, the sample was able to improve cell growth and improve survival rate compared with the negative control group (* *p* < 0.05). The results showed that esculetin has low-toxicity for HL-7702 cells in the selected concentration range.

### 2.3. Cytotoxic Effect of Esculetin for HepG2.2.15 Cells

**Figure 3:**
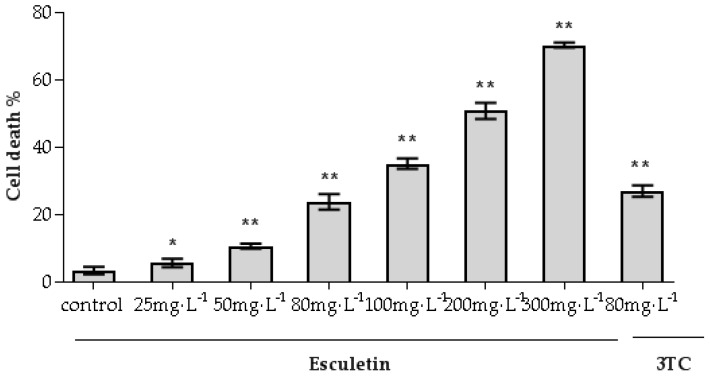
The cytotoxic effects of esculetin on HepG2.2.15 cells. The concentration of 50% cytotoxicity (CC_50_) was 217.48 mg/L (* *p* < 0.05, ** *p* < 0.01).

The cytotoxicity of esculetin for HepG2.2.15 cells was assessed using the trypan blue assay [25,26,27]. As shown in Figure 3, as the concentration of esculetin increased, the inhibitory effect of esculetin on HepG2.2.15 cells gradually increased. The concentration of 50% cytotoxicity (CC_50_) was 217.48 mg/L. These results were used to determine the esculetin dose range for subsequent experiments. In order to find the CC_50_ value, the cell experiment used such a large concentration rang, and the actual animal experiment was lower than this concentration. According to the concentration value of CC_50_, the sample concentration selected in the subsequent cell experiments was lower than the CC_50_.

### 2.4. Effects of Esculetin on the Secretion of the HBsAg and HBeAg in the HepG2.2.15 Cell Line

**Figure 4:**
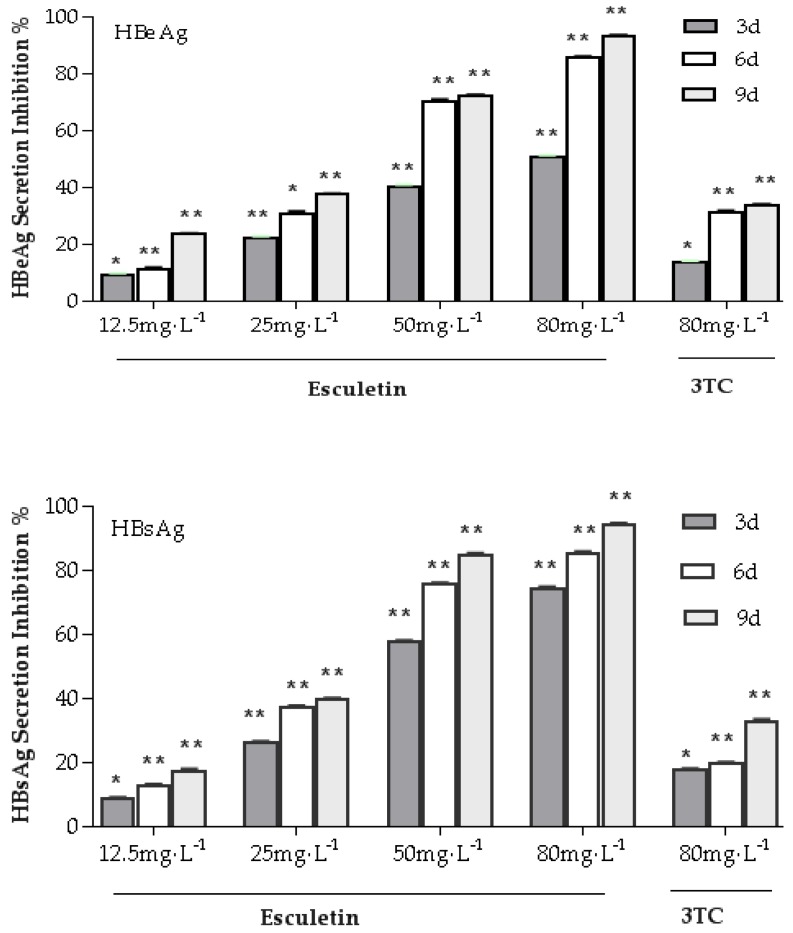
The inhibitory effects of esculetin on antigen secretion of HepG2.2.15 cell line. The cells were cultured at different concentrations of esculetin or 80 mg/mL of lamivudine (3TC) for 3 d, 6 d and 9 d, respectively. Moreover, the expression of HBsAg and HBeAg in the cell supernatant was detected using a specific enzyme-linked immunosorbent assay (ELISA) kit. The data are presented as means ± standard deviation (S.D) (n = 3) (* *p* < 0.05, ** *p* < 0.01).

As shown in Figure 4, treatment of HepG2.2.15 cells with various esculetin concentrations for 9 days resulted in significant reduction secretion of Hepatitis B surface antigen (HBsAg) and Hepatitis Be Antigen (HBeAg) in a time- and dose-dependent manner. Esculetin inhibited HBsAg production in HepG2.2.15 cells, with IC_50_ values of 43.12 mg/L at day 3, 29.59 mg/L at day 6 and 26.21 mg/L at day 9. Esculetin inhibited secretion of HBeAg in HepG2.2.15 cells, with IC_50_ values of 72.15 mg/L at day 3, 34.09 mg/L at day 6 and 27.32 mg/L at day 9. The results exhibited that esculetin could significantly reduce HBsAg and HBeAg levels compared to 3TC.

### 2.5. Effects of Esculetin on Secretion of Supernatant HBV DNA

**Figure 5:**
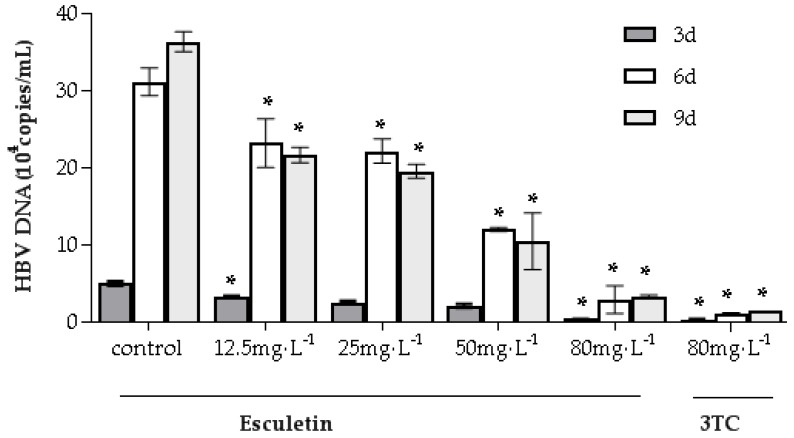
Inhibitory effects of esculetin on hepatitis B virus (HBV) DNA levels. For different periods, the cells were incubated at different concentrations of esculetin or 80 mg/L 3TC. The levels of HBV DNA in the supernatant were detected using fluorescent quantitative polymerase chain reaction (FQ-PCR). No-medication group was used as a negative control. The figures are presented as means ± S.D. (n = 3). * *p* < 0.05 compared to control (* *p* < 0.05, ** *p* < 0.01).

The effect of esculetin on HBV DNA expression in the supernatant of HepG2.2.15 cells was evaluated to further validate the anti-HBV activity of esculetin. According to the instructions of the fluorescent quantitative polymerase chain reaction (FQ-PCR) kit, the series of standard samples were tested. The standard curve has a good linear relationship, the correlation coefficient was 0.9954 and the formula for copy number was Cq = −3.28log_10_24 September 2019 (q) + 32.91. As can be seen from Figure 5, HepG2.2.15 cells were treated with 12.5 ~ 80 mg/L of esculetin or 80 mg/L of 3TC, and the level of HBV DNA decreased significantly. It also shows that esculetin has a dose-dependent inhibition of HBV DNA, but does not have a significant time-dependent effect. The activity of esculetin was similar to that of 3TC (80 mg/L) with a concentration of 80 mg/L, both showing strong inhibitory activity.

### 2.6. Effect of Esculetin on the Expression of HBx

**Figure 6:**
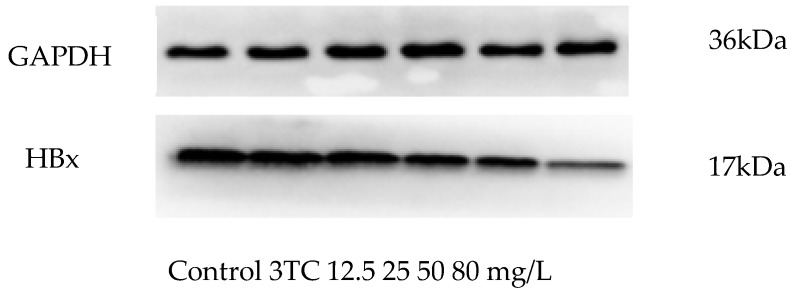
The level of expression hepatitis B virus X (HBx) protein in HepG2.2.15 cells: esculetin could reduce significantly the HBx protein expression, and its inhibitory was stronger than that of 3TC.

HBx protein is stably expressed in Hep G2.2.15 cell line, thus, it is used as an ideal test indicator for studying hepatitis B virus inhibitors [28]. In order to determine whether esculetin inhibits the HBx expression, the HBx protein was determined by western blot. As shown in Figure 6, esculetin could reduce significantly the HBx protein expression, and its inhibition by esculetin was stronger than 3TC.

### 2.7. Short-Term Toxicity of Esculetin on Ducklings

After 21 days of treatment, there was no significant difference in feather color, body weight, food intake, irritation response and mental state between the esculetin groups and the model control group. It demonstrates that esculetin had no obvious short-term toxicity for ducks.

### 2.8. Effect of Esculetin on Serum Content of DHBV DNA

**Figure 7:**
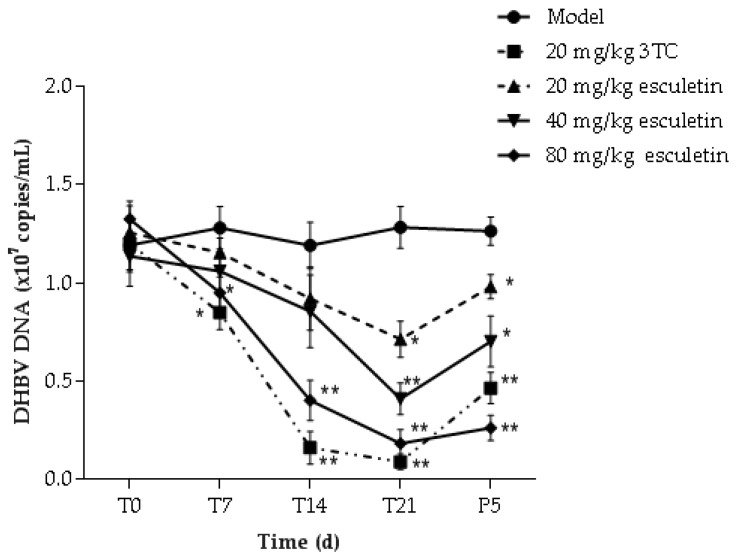
The effect of esculetin on DNA replication of serum duck hepatitis B virus. Serum from ducks was collected at different time points. Fluorescence quantitative PCR was used to detect DHBV DNA levels. The data is presented as the means ± S.D. (n = 8). * *p* < 0.05 compared to model group (* *p* < 0.05, ** *p* < 0.01).

The DHBV DNA levels in the serum of treated and untreated infected ducks were determined by FQ-PCR. The standard curve had an excellent linear relationship, the correlation coefficient was 0.9903, and the copy number was calculated as Cq = −3.32log_10_(q) + 34.62. The results of the copy number change of DHBV DNA in each group of serum are shown in Figure 7. After 21 days of treatment with esculetin and 3TC, DHBV DNA levels of each group were lower when compared to the model group (* *p* < 0.05). The high-dose esculetin treatment group on the inhibition effect of HBV DNA expression was close to 3TC. Five days after drug withdrawal (P5) in the 80 mg/kg esculetin treatment group, the degree of rebound of DHBV DNA was lower than that of the 3TC treatment group.

### 2.9. Effects of Esculetin on Serum DHBsAg and DHBeAg Levels

**Figure 8:**
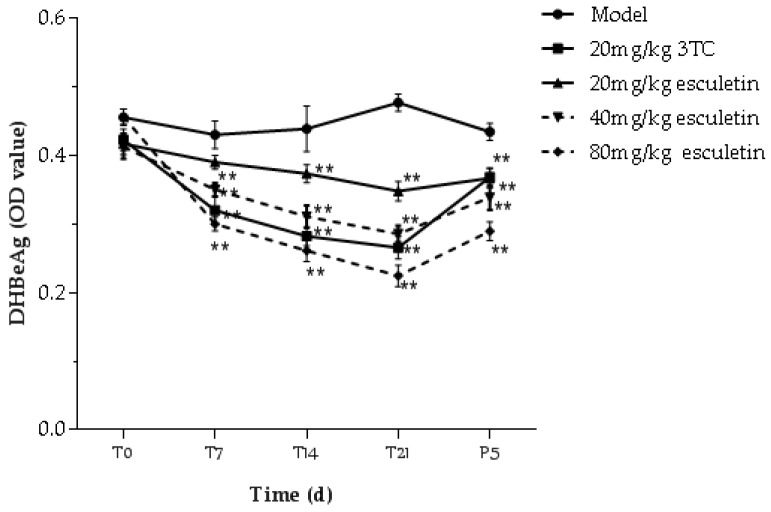

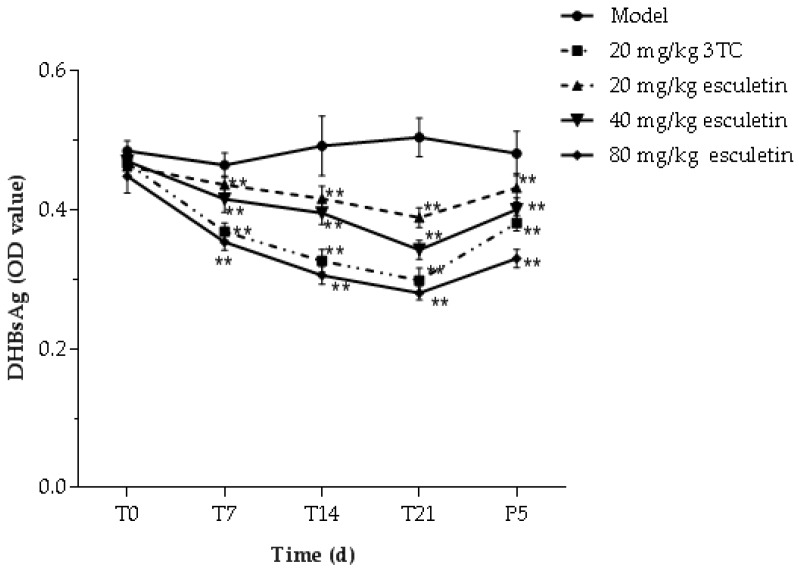
The inhibitory effect of esculetin on antigen serum levels. Serum was collected from ducks at different time points and the HBsAg and HBeAg levels were measured by ELISA kits. The data is presented as the means ± S.D. (n = 8). * *p* < 0.05 compared to the model group (* *p* < 0.05, ** *p* < 0.01).

Different concentrations of esculetin (20–80 mg/kg), 3TC (20 mg/kg) and saline acted on ducks once a day for 21 days, as shown in Figure 8. Before treatment, treatment for 7 days, 14 days, 21 days and after withdrawal of treatment for 5 days (P5), the presence of HBeAg and HBsAg in duck serum was detected. The results showed that HBeAg and HBsAg levels in the esculetin treatment groups were significantly lower than those in the model group. The 80 mg/kg esculetin treatment group inhibited the secretion of HBeAg and HBsAg more strongly than the 3TC group. In addition, on the fifth day after treatment, the rebound of HBeAg secretion in esculetin-treated ducks was less than that in the 3TC treatment group.

### 2.10. Analysis of ALT and AST Levels in Serum

As shown in Table 1 and Table 2, there was no significant difference in serum aspartate aminotransferase (AST) and alanine aminotransferase (ALT) levels between the treatment groups and the model group before treatment. After one week of treatment, the activities of ALT and AST in the 80 mg/kg esculetin-treated group and the 3TC-treated group were significantly decreased compared with the model group. At two weeks and three weeks of treatment, the levels of ALT and AST in the different concentrations of esculetin treatment groups decreased significantly, and there were significant differences compared with the model group.

### 2.11. Histopathological Examination of Duck Livers

**Figure 9:**
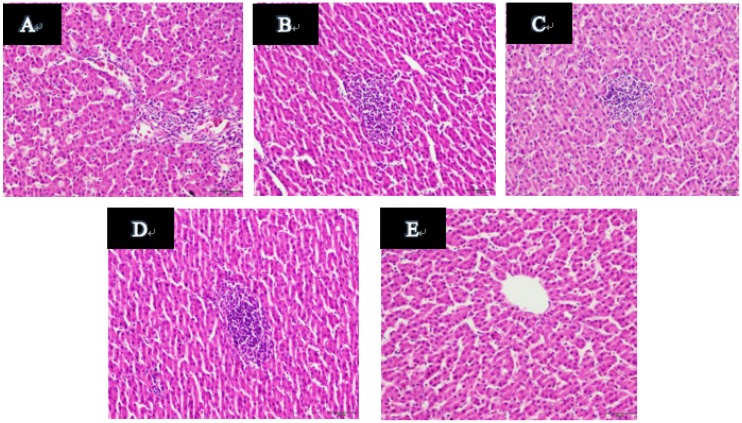
Results of pathological examination in duck liver tissue. A optical microscope (400×): (**A**) Model group; (**B**) 20 mg/kg 3TC; (**C**) 20 mg/kg esculetin; (**D**) 40 mg/kg esculetin; (**E**) 80 mg/kg esculetin.

A typical optical micrograph of a liver section is shown in Figure 9. The normal hepatocytes have a clear and regular structure, and the monolayers around the central vein are radially arranged. However, hepatocytes of the model control group showed inflammatory cell infiltration, ballooning degeneration and vacuoles degeneration (Figure 9A). Compared with the model group, the 3TC group showed significant improvement in cell degeneration, but there was still inflammatory cell infiltration (Figure 9B). Samples treated with esculetin exhibited dose dependent improvement of the hepatocellular architecture compared with the model group. Liver sections of the low-dose and medium-dose groups showed inflammatory cell infiltration, no ballooning degeneration (Figure 9C–D). In the meantime, liver sections of the high dose group showed no inflammatory cell infiltration and cell degeneration (Figure 9E).

Evaluation of the liver sections is mainly based on hepatocyte degeneration, necrosis and inflammatory cell infiltration. After esculetin treatment, the degree of necrosis and cell infiltration in the treatment group was better than that in the model group, and the above three parameters were dose-dependently improved. What is worth reminding is that in the high-dose group (80 mg/mL) these were more significant improvement compared with 3TC (20 mg/mL), and it also had a more significant liver-protecting effect.

## 3. Discussion

In this experiment, the HepG2.2.15 cell model was used to study the anti-HBV activity of esculetin from *Microsorium fortunei*. The results showed that CC_50_ of esculetin was 217.48 mg/L in the HepG2.2.15 cell. The expression of antigen in cell supernatant was detected by ELISA kits. The results indicated that esculetin can significantly inhibit the secretion of HBsAg and HBeAg at a non-toxic concentration range, and the inhibitory effect of esculetin on antigen was dose- and time-dependent. After the ninth drug treatment, the maximum inhibitory effect of esculetin on the secretion of HBsAg and HBeAg in the cell supernatant was 94.74% and 93.88%, respectively. The maximum inhibitory effect of 3TC on HBsAg and HBeAg was 41.58% and 47.92%, respectively, indicating that esculetin has a stronger inhibitory effect on secretion of HBsAg and HBeAg than 3TC, and has lower cell toxicity. In addition, real-time PCR was used to detect the level of HBV DNA in the supernatant, and the results showed that esculetin significantly reduced the expression of HBV DNA in the supernatant of the cells compared with the negative control group. The inhibitory effect of esculetin on HBV DNA was enhanced with increasing the drug concentration and treatment time. Furthermore, HBx protein was detected by western blot and the esculetin significantly inhibited the expression of HBx protein, and its inhibitory effect was stronger than with 3TC. This result further proves that esculetin may have anti-HBV activity in vitro.

To further confirm that esculetin has anti-HBV activity, this study also used the animal model of Guangxi congenital infection of DHBV to study the anti-HBV activity of esculetin in vivo, which is a standard model for screening anti-hepatitis B virus drugs over the world [29,30]. Experimental results in vivo showed that esculetin significantly inhibited the replication of DHBV, and its efficacy was positively correlated with therapeutic dose and treatment time. After the 14th treatment, compared with the model group, the esculetin groups significantly reduced the levels of HBsAg and HBeAg in serum, and 80 mg/kg of esculetin and 20 mg/kg of 3TC significantly reduced expression of DHBV DNA. On the 21st day of treatment, each group of esculetin was able to significantly reduce HBsAg, HBeAg and DHBV DNA levels. Even after five days of drug withdrawal, the levels of the above indicators were still lower than those of the model group. This suggests that esculentine effectively inhibits the replication of DHBV in the body. The results indicate that esculetin can remarkably suppress the DHBV replication in vivo.

In addition, in order to study the protective effect of esculetin on liver injury caused by HBV, levels of ALT and AST in the serum and pathological changes in the liver were also evaluated. On the 14th and 21st day of treatment, and P5, esculetin significantly reduced the enzyme activity of ALT and AST compared to the model group. Furthermore, the result of the histopathological examination demonstrated that the hepatocellular architecture of the esculetin groups (80 mg/kg) was more significantly improved compared to the 3TC group.

**Figure 10:**
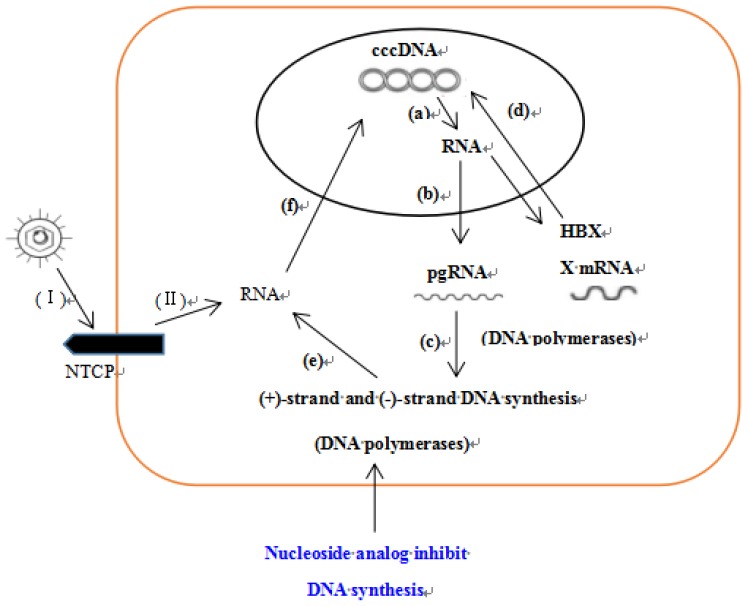
Anti-HBV mechanism of nucleoside analogues. (I). Adhesion and invasion. (II). Removal of nucleic capsid. (**a**) Viral RNA transcription. (**b**) RNA nuclear export. (**c**) DNA polymerases synthesize positive and negative strands. (**d**) Regulation of covalently closed circular deoxyribonucleic acid by hepatitis B virus X protein. (**e**) Messenger Ribonucleic Acid (mRNA) formation and (**f**) nuclear transport of mRNA.

Many anti-HBV drugs, such as interferons, nucleotide analogs, etc., have been used clinically, but they have certain side effects. Therefore, it is urgent to find new anti-HBV drugs. In our study, we found that both 3TC and esculetin can suppress the antigen secretion and DNA replication of HBV, and the inhibitory effect of HBeAg and HBsAg were higher than 3TC in efficacy. It has been reported that 3TC can significantly inhibit HBV-DNA synthesis by reducing the biological activity of the DNA polymerase dependent on HBV RNA [31,32] (Figure 10). This provides an idea for studying the anti-HBV mechanism of esculetin. The results showed that esculetin reduces the levels of HBV DNA and DHBV DNA, and inhibits the expression of HBx protein. HBx protein plays a key role in the formation of Hepatocellular Carcinoma, and it can affect viral replication, cell proliferation and differentiation and normal hepatocyte infection. Based on the results, we hypothesized that esculetin may exert anti-HBV effects by inhibiting DNA synthesis, affecting HBV DNA replication, and down-regulating HBx protein expression, which in turn even leads to cell death. In addition, esculetin has multiple pharmacological activities, e.g., antitumor [33,34,35], antioxidation [36], antibacterial [37], etc. It should also be further developed as an anti-HBV drug.

## 4. Materials and Methods

### 4.1. Chemicals

Dulbecco’s modified Eagle medium (DMEM) was purchased from Solebo Biotechnology Co., Ltd. (Beijing, China). 3TC was obtained from Yuanye Bio-Technology & Co Ltd. (Shanghai, China). HBsAg and HBeAg kits were from Kehua Bio-Engineering & Co Ltd. (Shanghai, China). Fluorescence quantitative PCR (FQ-PCR) kit was purchased from DAAN Gene & Co Ltd., Of Sun Yat-sen University (Guangzhou, China). Rabbit anti-HBx (1:500), Rabbit anti-reduced glyceraldehyde-phosphate dehydrogenase (GAPDH) (1:5000), Horseradish peroxidase-conjugated goat anti-rabbit IgG antibody (goat-anti-rabbit IgG-HRP)(1:5000) were obtained from Absin Bioscience Co Ltd. (Shanghai, China). Bicinchoninic acid protein assay (BCA) kits were products of Beyotime Biotechnology (Shanghai, China). DHBsAg, DHBeAg, DHBV FQ-PCR kits and one-tube virus DNAout kits were supplied by LMAI Bio-Engineering & Co Ltd. (Shanghai, China). AST and ALT reagent kits were obtained from Jiancheng Bioengineering Research Institute (Nanjing, China).

### 4.2. General Experimental Instruments

NMR spectra were analyzed on a Bruker DRX-500 MHz spectrometer (Bruker Optics, Ettlingen, Germany). Semi-preparative HPLC was carried out on an Agilent 1260 liquid chromatography (LC) with a Zorbax SB-C_18_ column (5 μM, 9.4 mm × 250 mm) (Agilent, Santa Clara, CA, USA). Column chromatography (CC) was performed on silica gel (100–200 or 200–300 mesh, Qingdao Marine Chemical, Inc., Qingdao, China), MCI gel (75–150 μM, Mitsubishi Chemical Corporation, Tokyo, Japan), Sephadex LH-20 (Amersham Pharmacia Biotech, Uppsala, Sweden), and Lichroprep RP-18 gel (40–60 μM, Merck, Darmstadt, Germany).

### 4.3. Plant Material

The leaves and stems of *Microsorium fortunei* (40.0 kg) were collected in Guangxi Resources County of Guangxi Province, China, in 2015, and esculetin was identified by Prof. Yunqiu Li, College of Pharmacy, Guilin Medical University. The voucher specimen (No. 2016101201) has been deposited in the College of Pharmacy, Guilin Medical University, China.

### 4.4. Extraction and Isolation

The powdered dried leaves and stems of *Microsorium fortunei* (40.0 kg) were extracted three times with 75% ethanol (each 1 h). The extract was concentrated under reduced pressure to a non-alcoholic taste. The concentrated extract was separately extracted with ethyl acetate to obtain one extracting component, that is, an ethyl acetate component (978 g). The ethyl acetate extract was separated by silica gel column chromatography, and eluted successively (1:0, 9:1, 8:2, 2:1, 1:1, 0:1, CHCl_3_–MeOH) to obtain six fractions Fr.A ~ F. The elution fraction (9:1, CHCl_3_–MeOH) was separated by MCI column chromatography, decolorized by 90%, and separated by gel column. After TLC detection, the same components were combined to obtain Fr.I ~ Fr.V. The Fr.IV (8.0 g) was further separated by RP-18 column chromatography, and gradient elution was carried out (0% ~ 100%, MeOH-H_2_O) to obtain a total of 29 fractions of Fr.IV.1 ~ IV.29. The Fr.IV.7 component (1.0 g) was separated by RP-18 column and eluted with 15% methanol water for 1 h to obtain Fr.IV.7.1-Fr. IV.7.5. The fractions of Fr.IV.7.2 (0.2 g) were separated and purified by HPLC, and the compound, esculetin (23.0 mg, t_R_ = 15.479 min) was separated (10% ~ 100% MeOH-H_2_O, 1 mL/min) [38]. Its structure is shown in Figure 1.

### 4.5. Cell Culture

The HepG2.2.15 cell line and HL-7702 cell line were provided by Beijing 302 hospital. These two cells were incubated in DMEM with 10% fetal calf serum, and were screened periodically with 380 μg/mL G418 at 37 °C in a 5% CO_2_ and 100% humidity incubator. All of cells in the flask were digested with 0.25% trypsin (1 mmol/L ethylene diamine tetraacetic acid, EDTA), which were resuscitated for carrying out further experiments [39].

### 4.6. Cytotoxicity Assay on HL-7702 Cells

The HL-7702 cell line is a normal liver cell line. In short, HL-7702 cells (2 × 10^5^ cells/mL) were inoculated in culture dishes. After 24 h of incubation, the cells were treated with esculetin (12.5, 25, 50, 100, 200 mg/L) and Silybin (positive control ([40,41]) for 24 h. Then, the cells were collected and prepared in cell suspension (1 × 10^6^ cells/mL). The cell suspension and the 0.4% trypan blue solution were mixed in a ratio of 1:1. The mixture was gently mixed at room temperature and allowed to stand for 5 min. Then, 10 μL of the mixture was taken for cell counting.

Cells viability (%) = (Number of viable cells/Total number of cells) × 100% (1)

### 4.7. Cytotoxicity Assay on HepG2.2.15 Cells

The cytotoxic effect of the esculetin on HepG2.2.15 was assessed using the trypan blue assay [25,26,27]. HepG2.2.15 cells were inoculated in culture dishes (2 × 10^5^ cells/mL). After incubation for 24 h, the supernatant was replaced with DMEM containing different concentrations of esculetin (25, 50, 80, 100, 200, 300 mg/L). The media was refreshed every two days. After treatment for nine days, the supernatant was collected. Then, the cells were collected by trypsinization and made into a 1 × 10^6^ cells/mL cell suspension. The mixture was subjected to cell counting in the same manner as described above.

Cells death (%) = [1 − (Number of viable cells/Total number of cells)] × 100%(2)

### 4.8. Detection of HBsAg and HBeAg in the Supernatant by ELISA

HBsAg and HBeAg expression levels in the supernatant were detected in terms of the ELISA kits’ protocol. Read the absorbance of the plate directly at 450 nm with a microplate reader.

Antigen inhibition rate (%) = (OD_control_ − OD_sample_)/OD_control_ × 100% (3)

### 4.9. Determination of HBV DNA in the Supernatant by FQ-PCR (Fluorescent Probe Method)

The HBV DNA in the supernatants was detected by FQ-PCR. HBV DNA was amplified using small-scale real-time PCR system. The reaction procedure of FQ-PCR was as follows: initial denaturation at 93 °C for 2 min, followed by 10 cycles of denaturation at 93 °C for 45 s, annealing at 55 °C for 60 s, extension by 30 cycles of denaturation at 93 °C for 30 s, annealing at 55 °C for 45 s. Standard solution in the kit was used to prepare the standard curve [42,43].

### 4.10. Determination of HBx Protein by Western Blot

The HBx protein was evaluated by using an improved method of reference [44]. HepG2.2.15 cells were lysed with a radio-immunoprecipitation assay (RIPA) buffer buffer after treatment with esculetin (12.5, 25, 50, 80 mg/L) and 3TC (100 mg/L) for 72 h, and the protein was quantified with a BCA kit. The membrane was blocked in 5% skim milk for 2 h, and incubated overnight at 4 °C with rabbit anti-HBx (1:500) and rabbit anti-GAPDH (1:5000). After washing with tris buffered saline tween (TBST) five times for 10 min each time, the membranes blocked in 5% skim milk for another 30 min. The polyvinylidene fluoride (PVDF) membrane was incubated with a secondary antibody (goat-anti-rabbit IgG-HRP, 1:5000) at room temperature for 1.5 h, then washed with TBST five times (5 min each time). The protein bands were illuminated by a chemiluminescent gel imaging system.

### 4.11. Short-Term Toxic Reaction of Esculetin on Ducklings

Slightly modified from the method of Huang et al. [45], short-term toxicity studies were conducted in ducks. Fifty ducks of the congenitally infected DHBV were split freely into five groups (10 ducklings/per group) with high-, middle- and low-doses of esculetin, positive drug 3TC and saline, and treated for 21 days. Animal vital signs including body weight, gait, food intake, feather color, health status and response to stimuli were supervised in the course of the tests.

### 4.12. Animals and Treatments

Ducklings infected with the hepatitis B virus were purchased from Guilin Wenshi Group Co., Ltd. (Guangxi, China). 200 one-day-old ducks were purchased, including both male and female ducks. Fifty ducks (serum viral load 10^6^–10^8^ copies/L) infected with DHBV were randomly divided into five groups by FQ-PCR: high-, middle- and low-dose groups of esculetin (80, 40, 20 mg/kg), 3TC group (20 mg/kg) and model control group (saline). The ducks were intragastrically treated for 21 days. On the day before the treatment, on the 7th, 14th and 21st days of treatment and the 5th day after treatment, serum samples were collected and stored at −80 °C for analysis. At the end of the experiment, the liver tissue of the duck was submitted to pathological examination. The animal experiment section was supervised by the Animal Care and Welfare Committee of Guilin Medical University, which approved all operations in the animal experiments.

### 4.13. Measurement of Serum DHBV DNA by FQ-PCR Assay

The viral DNA in the duck serum was extracted using Viral DNA Extraction Kits. DHBV DNA was detected with FQ-PCR kits. DHBV DNA was amplified using small-scale real-time PCR system. The forward primer: 5′-CCAACACATGGCGCAATATC-3′, the reverse primer: 5′-GCCTAAAGGTATCTCCTCAG-3′. The reaction conditions of FQ-PCR were as follows: denaturation at 95 °C for 1 min, followed by 30 cycles of denaturation at 95 °C for 15 s, annealing at 60 °C for 15 s, extension at 72 °C for 15 s. The standard solution in the kit was used to make a standard curve [46,47].

### 4.14. Measurement of Serum DHBsAg and DHBeAg

HBsAg and HBeAg in the serum were measured using ELISA kits (LMAI Bio-Engineering Co., Ltd., Shanghai. China) in terms of its protocol. The plates were read directly at 450 nm with a microplate reader.

### 4.15. Analysis of Serum ALT and AST

Serum samples from each group were collected on days 0, 7, 14, 21 and P5. Changes of AST and ALT in duck serum were analyzed by Transaminase kits (Nanjing Jiancheng Bioengineering Research Institute, Nanjing, China).

### 4.16. Histopathological Examination of the Duck Livers

On P5, the right lobes were fixed with 4% paraformaldehyde and sent to the pathology department of the Affiliated Hospital of Guilin Medical College for pathological section, including paraffin embedding, sectioning, staining (hematoxylin-eosin staining), and observation of pathological changes by optical microscopy.

### 4.17. Statistical Analysis

All data were statistically analyzed using SPSS 18.0 software. Mean values of each group were compared by one-way analysis of variance (ANOVA). Data were expressed as means ± standard deviation (S.D.). Under the condition of *p* < 0.05, the differences were considered to be statistically significant.

## Figures and Tables

**Table 1:** Effect of esculetin on serum alanine aminotransferase (ALT).

Group	Concentration (mg/kg)	ALT				
		T0	T7	T14	T21	P5
**Model**		89.67 ± 4.28	83.53 ± 5.5	85.25 ± 6.83	79.13 ± 3.35	77.00 ± 7.72
3TC	20	86.35 ± 5.39	62.95 ± 7.36 **	47.88 ± 4.47 **	39.45 ± 6.00 **	65.65 ± 5.42
	80	77.97 ± 6.63	56.76 ± 4.16 **	45.56 ± 3.60 **	36.08 ± 7.59 **	49.99 ± 8.56 **
esculetin	40	85.57 ± 5.92	68.73 ± 7.23 *	58.88 ± 7.82 **	48.85 ± 6.31 **	60.87 ± 4.91 **
	20	78.88 ± 4.91	75.59 ± 6.53	69.31 ± 9.06 *	65.20 ± 7.09 *	72.02 ± 7.02

Data is expressed as means ± S.E. Compared with the model group: * *p* < 0.05, ** *p* < 0.01.

**Table 2:** Effect of esculetin on serum aspartate aminotransferase (AST).

Group	Concentration (mg/kg)	AST				
		T0	T7	T14	T21	P5
**Model**		160.65 ± 17.38	163.18 ± 14.49	162.07 ± 13.14	167.24 ± 15.05	150.44 ± 11.20
3TC	20	175.98 ± 13.79	120.79 ± 12.92 **	94.86 ± 13.05 **	80.70 ± 12.11 **	110.40± 12.12 **
	80	156.93 ± 14.72	114.04 ± 13.34 **	89.19 ± 11.74 **	70.52 ± 13.32 **	89.72 ± 14.55 **
esculetin	40	165.83 ± 13.58	137.95 ± 10.88 *	118.48 ± 15.59 **	109.74 ± 13.88 **	126.94 ± 15.92 **
	20	169.19 ± 14.00	146.01 ± 14.36 *	130.77 ± 14.54 **	117.08 ± 10.48 **	142.44 ± 12.43

Data is expressed means ± S.E. Compared with the model group: * *p* < 0.05, ** *p* < 0.01.

## Supplementary Materials

The following are available online. Figure S1: HPLC of esculetin; Figure S2: ^1^H-NMR spectrum of esculetin (DMSO-*d*_6_, 500 MHz); Figure S3: ^13^C-NMR spectrum of esculetin (DMSO-*d*_6_, 125 MHz).
