# Supplementary material for: Anti-Hepatitis B Virus Activity of Esculetin from Microsorium fortunei In Vitro and In Vivo
Source: Molecules. 2019 Sep 25;24(19):3475. doi: 10.3390/molecules24193475 (PMC6803987; doi:10.3390/molecules24193475)
Supplement: Supplementary file 1 [file molecules-24-03475-s001.pdf]

Supplementary Information

Anti-Hepatitis B Virus Activity of Esculetin from *Microsorium fortunei* In Vitro and In Vivo

Si-Xin Huang[1,+], Jun-Fei Mou[2,+], Qin Luo[3], Qing-Hu Mo[1], Xian-Li Zhou[2,*], Xiao Huang[1], Qing Xu[4], Xiang-Duan Tan[1], Xu Chen[1], Cheng-Qin Liang[1,]**

1 College of Pharmacy, Guilin Medical University, Guilin 541004, Guangxi, China; huangsixin@stu.glmc.edu.cn (S.-X. H.); moxqxom@163.com (Q.-H. M.); gyhuangxiao@163.com (X. H.); tandy@glmc.edu.cn (X.-D. T.); chenxu@glmc.edu.cn (X.C.); cqliang@glmc.edu.cn (C.-Q. L.)

2 Biotechnology Institute, Guilin Medical University, Guilin 541004, Guangxi, China; qiaoyifei@stu.glmc.edu.cn (J.-F.M.); xlzhou2019@glmc.edu.cn (X.-L. Z.)

3 Science Experiment Center, Guilin Medical University, Guilin 541004, Guangxi, China; luoqin5020@glmc.edu.cn(Q. L.)

4 Guangxi Key Laboratory of Molecular Medicine in Liver Injury and Repair, Guilin Medical University, Guilin 541004, Guangxi, China; xq5895801@163.com (Q. X.)

* Correspondence:. cqliang@glmc.edu.cn (C.-Q.L.); xlzhou2019@glmc.edu.cn (X.-L. Z.);
Tel:+0773-5891498 (C.-Q.L.); +0773-3680722 (X.-L. Z.);
Fax:+0773-5894158 (C.-Q.L.); +0773-3680722 (X.-L. Z.)

Figure S1. High performance liquid chromatogram of esculetin.

Figure S2. ^{1}H-NMR of esculetin (DMSO-d_6, 500 MHz).

Figure S3. ^{13}C-NMR of esculetin (DMSO-d_6, 125 MHz).

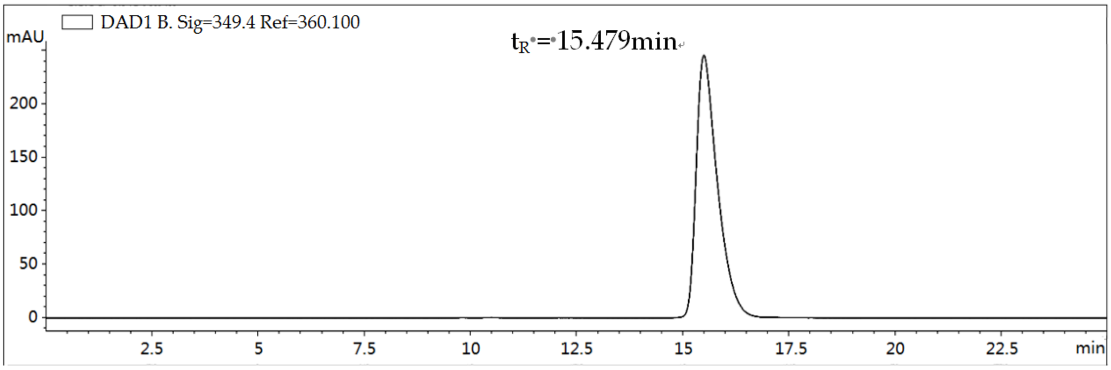

Figure S1. HPLC of esculetin: Wave length = 349 nm; Flow rate = 1 mL/min; Column temperature = 36℃; Mobile phase: Acetonitrile :0.5% acid water = 7:93; t_R = 15.479min.

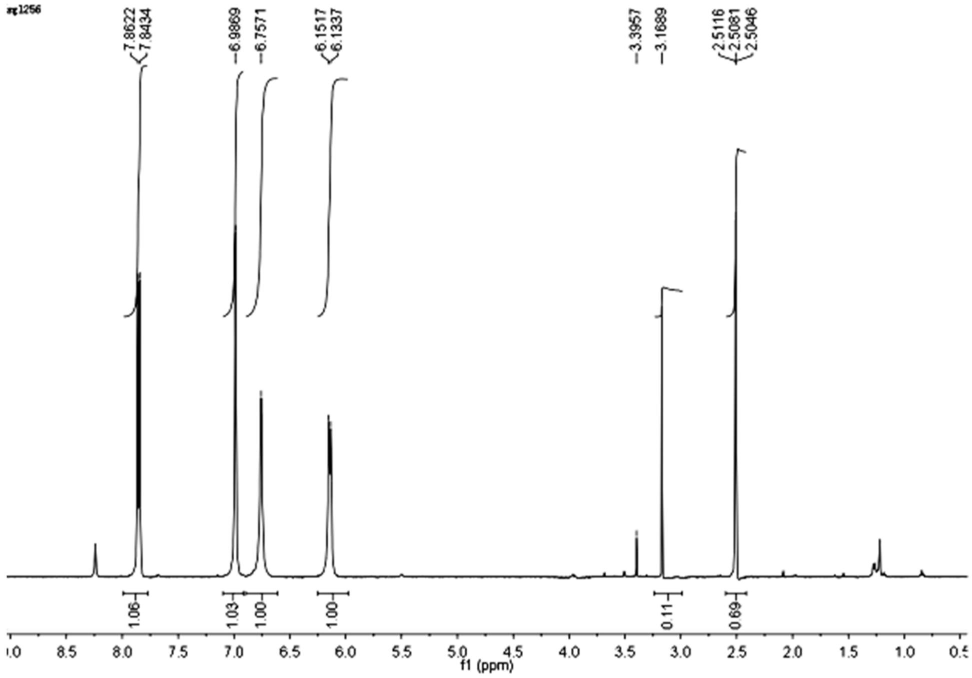

Figure S2. ^{1}H-NMR of esculetin (DMSO-d_6, 500 MHz).

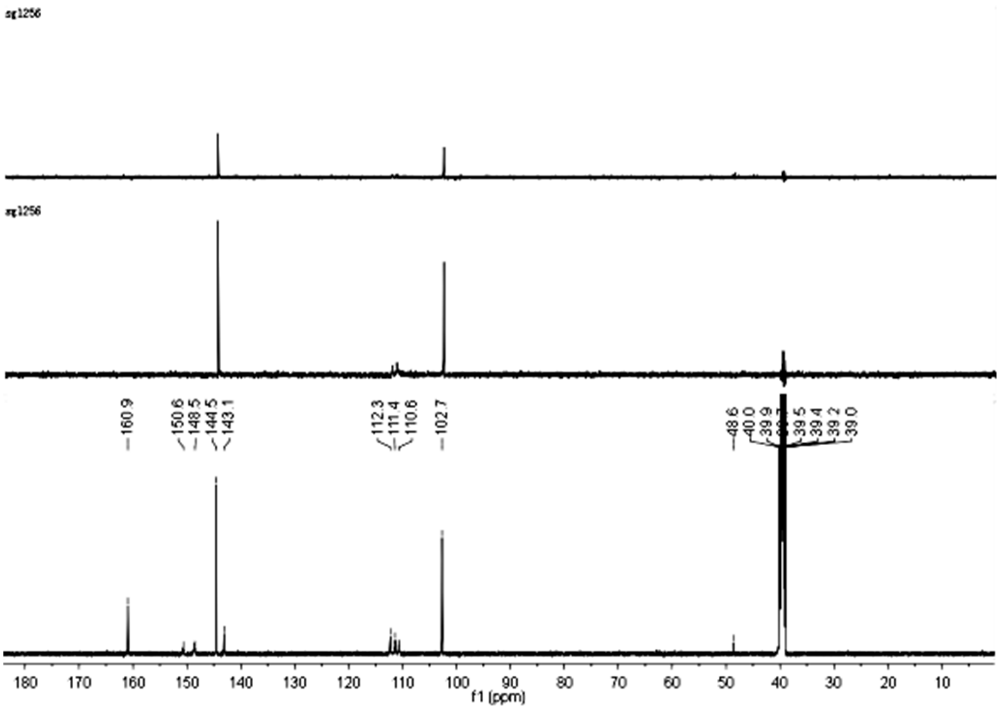

Figure S3. ^{13}C-NMR of esculetin (DMSO-d_6, 125 MHz).
